# Amyloid-mediated remineralization for tooth hypoplasia of cleidocranial dysplasia

**DOI:** 10.3389/fcimb.2023.1143235

**Published:** 2023-03-03

**Authors:** Xiaohe Guo, Xiaoxue Yang, Peisheng Liu, Xiaoyao Huang, Yang Gu, Hao Guo, Kun Xuan, Anqi Liu

**Affiliations:** ^1^ State Key Laboratory of Military Stomatology and National Clinical Research Center for Oral Diseases and Shaanxi Clinical Research Center for Oral Diseases, Department of Preventive Dentistry, School of Stomatology, Fourth Military Medical University, Xi’an, Shaanxi, China; ^2^ Department of Stomatology, The 985 Hospital of Chinese People's Liberation Army (PLA), Taiyuan, Shanxi, China

**Keywords:** cleidocranial dysplasia, stem cells from human exfoliated deciduous teeth, tooth hypoplasia, caries prevention, amyloid, biomineralization

## Abstract

**Introduction:**

Cleidocranial dysplasia (CCD) is an autosomal-dominant, heritable skeletal and dental disease, involving hypoplastic clavicles, defective ossification of the anterior fontanelle, dentin and enamel hypoplasia, and supernumerary teeth, which can seriously affect the oral and mental health of patients. Amyloid-like protein aggregation, which is established by lysozyme conjugated with polyethylene glycol (Lyso-PEG), forms a mineralized nanofilm layer on a healthy enamel surface. However, whether it can form a remineralization layer in dental tissues from CCD remains unclear.

**Methods:**

This study evaluated deciduous teeth from healthy individuals and a patient with CCD. Because pulp and dentin are functionally closely related, stem cells from human exfoliated deciduous teeth (SHED) from CCD patients and healthy individuals were collected to compare their biological properties.

**Results:**

The results found that deciduous teeth from patients with CCD exhibited dentin hypoplasia. In addition, the proliferative ability and osteogenic potential of SHED from patients with CCD were lower than those of control individuals. Finally, Lyso-PEG was applied to dentin from the CCD and control groups, showing a similar remineralization-induced effect on the dentin surfaces of the two groups.

**Conclusion:**

These results extend our understanding of the dentin and SHED of patients with CCD, exhibiting good caries-preventive capacity and good biocompatibility of Lyso-PEG, thus providing a novel dental therapy for CCD and patients with tooth hypoplasia.

## Introduction

Cleidocranial dysplasia (CCD) is an autosomal dominant heritable skeletal disease that affects cranial sutures, teeth, and clavicles, and the putative causative gene is Runt-related transcription factor 2 (*RUNX2*) (Xuan et al., 2010; Hordyjewska-Kowalczyk et al., 2019; Komori, 2020). Typical clinical dental features include skeletal class III tendency, tooth hypoplasia, delayed eruption of the permanent dentition, and multiple impacted supernumerary teeth (Shih-Wei Cheng et al., 2022). Currently, therapeutic strategies for CCD are still based on symptomatic treatment. Studies have reported that CCD teeth exhibit insufficient mineralization of enamel and dentin, leading to multiple caries and early tooth loss (Chang et al., 2017). However, compared with abnormal dental replacement and multiple supernumerary teeth, tooth hypoplasia, which also influences the oral and mental health of individuals, has received less attention. Further investigation will extend our understanding of tooth hypoplasia in patients with CCD, along with promoting oral health.

Stem cells in dental pulp participate in dentin formation and repair that play a critical role in maintaining tooth homeostasis (Huang et al., 2021; Jung et al., 2022). Stem cells from human exfoliated deciduous teeth (SHED) are mesenchymal stem cells (MSCs) that exhibit proliferation and multilineage differentiation properties in dental pulp (Miura et al., 2003). Exploring the function of SHED in patients with CCD would be beneficial for understanding dentin hypoplasia. With rapid developments in the field of materials, extensive efforts have been focused on establishing ideal materials to prevent tooth decay and enhance the level of oral health. In a previous clinical trial, our research group applied amyloid-like oligomeric nanoparticles, which were established using lysozyme conjugated with polyethylene glycol (Lyso-PEG), on a healthy enamel surface to obtain a favorable remineralization layer (Yang et al., 2022). However, whether Lyso-PEG emulsion induces dentin remineralization remains to be elucidated. Investigating these factors is beneficial to prevent tooth caries and prolong the lifetime of the tooth in patients with CCD and tooth hypoplasia.

In this study, a family with CCD and a novel missense mutation in *RUNX2* was investigated. The properties of the tooth and SHED of the patients with CCD were examined. Then, a Lyso-PEG emulsion-treated tooth slab was used to analyze the caries preventive capacity. This study provides more information about the dentin and SHED of CCD, and is the first to provide a material, Lyso-PEG emulsion, for caries prevention in patients with CCD.

## Materials and methods

### Clinical examination and mutation analysis

The proband (8 years old) with a clinical diagnosis of CCD and her family participated in the current study. All subjects were free of clinical evidence of recent infection. Informed consent was obtained from all individuals in accordance with a protocol approved by the Institutional Review Board and the Ethics Committee of the Fourth Military Medical University (FMMU).

Blood samples (5 ml) were collected and stored in anticoagulant tubes containing ethylenediaminetetraacetic acid (EDTA). Genomic DNA was extracted with the use of a genomic DNA purification kit (version 3.0, Baosheng Company, China) according to the manufacturer’s instructions. DNA sequences were analyzed using the BLAST nucleotide program (http://www.ncbi.nlm.nlh.gov/BLAST).

### Tooth morphology and structure observation

After obtaining written consent from all participants, we extracted the retained deciduous teeth from healthy individuals (control group) and the proband with CCD (CCD group) as experimental samples at the School of Stomatology, FMMU. The crowns were cleaned using a rotating prophylactic brush until the plaque colorant indicated no attachment. The tooth samples were sectioned perpendicular to the dental crown using a slow-speed diamond saw, then ground and polished using 400-, 800-, 1500-, and 2000-grit silicon carbide abrasive paper lubricated with water to flatten the outer tooth surface and finally stored in a thymol solution (0.2 wt %) at 4°C.

To observe the structure and mineralization of the teeth, the tooth slabs were observed using a stereo microscope (DM2500; Leica, Germany), a scanning electron microscope (SEM; S4800; Hitachi, Japan), and Energy dispersive X-ray analysis (EDX; Model 550i; IXRF Systems, USA). The samples were dried by ethanol gradient dehydration (30, 50, 60, 70, 80, 90, 100, and 100 and 100%) for 10 min per step. The tooth slab samples were dried and mounted on an aluminum stage with carbon tape. The surfaces of the tooth slabs were sputtered with Pt and observed at an accelerating voltage of 5 kV. Both coronal and cross-sectional views of the sectioned dentin samples were observed using SEM. The EDX spectra were analyzed using the same samples.

### Isolation and culture of SHED

SHED from healthy individuals (control group) and proband (CCD group) were isolated and cultured from the dental pulp of deciduous teeth using the collagenase digestion method and cultured in α-minimum essential medium (MEM) containing 10% fetal bovine serum (FBS), as previously described (Xuan et al., 2018). SHED derived from three young clinically healthy male donors of similar ages were used as controls. SHED from the CCD were used as the test group. When primary SHED proliferated to 80%–90% confluence, the adherent cells were digested with 0.25% trypsin (MP Biomedicals, USA) and passaged *in vitro*. Third and fourth-generation SHED were used in the experiments.

### 5-ethynyl-2′-deoxyuridine (EdU) assay

Fourth-generation SHED from control and CCD groups were cultured on the base of carry sheet glass in a 24-well plate at an initial seeding density of 2000 cells per well for 24 h. Cell proliferation was analyzed using the EdU assay (kFluor488-EdU; KeyGEN BioTECH, China) after culturing for 24 h, according to the manufacturer’s instructions. The samples were then viewed using a confocal laser-scanning microscope (CLSM). Excitation/emission wavelengths of 350/495 nm and 461/520 nm were used for EdU and Hoechst staining, respectively.

### Cell Counting Kit-8 (CCK-8) analysis

Fourth-generation SHED from control and CCD groups were cultured in a 96-well plate at an initial seeding density of 5000 cells per well. Cell proliferation was analyzed using the CCK-8 (Beyotime, China) after culturing the different groups for 7 d. At each predetermined time point (Days 1, 4, and 7), 10 μl of the CCK-8 solution was added to each well and incubated for 4 h at 37°C. The optical density (OD) was measured using a microplate reader (Bio-Rad 680; Bio-Rad, USA) at a wavelength of 450 nm, and the assay was repeated five times.

### Immunofluorescent staining

Fourth-generation SHED from control and CCD groups were cultured on round coverslips (14 × 14 mm) at an initial seeding density of 1000 cells per well. After culturing for 24 h, round coverslips were fixed with 4% paraformaldehyde at 4°C for 30 min. Round coverslips were permeabilized with 0.3% Triton X-100 (Sigma-Aldrich, 93443) for 15 min and subsequently blocked using 5% normal goat serum at 37°C for 30 min. The coverslips were incubated with anti-RUNX2 primary antibody (Cell Signaling, #12556, rabbit, 1:200 dilution) overnight at 4°C and then incubated with Cy3-conjugated anti-rabbit secondary antibody (Cy3 AffiniPure Goat Anti-Rabbit IgG [H+L], 1:400 dilution) at 4°C for 2 h. Nuclear counterstaining was performed with Hoechst 33342 (Sigma-Aldrich, 14533) at 4°C for 10 min. For cytoskeleton immunostaining, cells were permeabilized with 0.2% Triton X-100 in phosphate-buffered saline (PBS) for 10 min and then treated with 5% bovine serum albumin (BSA) in Tris-buffered saline (TBS) for 30 min. F-actin was stained with fluorescein-phalloidin solution (Molecular Probes) (Med Chem Express, HY-K0902) for 10 min at 4°C, and the nuclei were labeled blue by incubation with 4′,6-diamidino-2-phenylindole (DAPI; BD Pharmingen, 564907, 1:200 dilution) for 10 min. Excitation/emission wavelengths of 350/460 and 488/530 nm were used for DAPI and F-actin, respectively. Images were captured using a CLSM (Nikon, Japan).

### Alkaline phosphatase (ALP) and Alizarin Red S staining

Fourth-generation SHED from control and CCD groups were analyzed for their capacity to differentiate into osteogenic lineages. Cells were seeded in six-well plates at a density of 2 × 10^5^ cells per well. When the cells reached 70% confluence, the culture medium was replaced with osteogenic medium containing 50 μg/ml ascorbic acid (MP Biomedicals), 2 mM β-glycerophosphate (Sigma-Aldrich), and 10 nM dexamethasone (Sigma-Aldrich). After 7 d of osteogenic induction, ALP staining was performed using an alkaline phosphatase assay kit (P0321; Beyotime, China) according to the manufacturer’s protocol, to evaluate ALP activity. After 14 d, Alizarin Red S staining was performed to assess the extent of mineralization. Briefly, the cells were fixed using 4% paraformaldehyde for 30 min, washed with PBS, stained with Alizarin Red S solution for 30 min, and then washed again with PBS.

### Oil Red O staining

Fourth-generation SHED from control and CCD groups were seeded in six-well plates at a density of 2 × 10^5^ cells per well. When the cells reached 70% confluence, the culture medium was replaced by adipogenic induction medium containing 0.5 mM 3-isobutyl-1-methylxanthine, 1 μM dexamethasone, and 0.1 mM indomethacin (Sigma-Aldrich). After the 14-day adipogenic induction, the cells were fixed using 4% paraformaldehyde for 30 min, washed using PBS, stained with Oil Red O (Aladdin, China) solution for 30 min, and then washed again with PBS.

### Real-time quantitative reverse transcription -polymerase chain reaction (qRT-PCR)

Total RNA was extracted from the samples using TRIzol reagent (Invitrogen), and reverse-transcribed into cDNA. Expression of osteogenic and adipogenic markers, including *ALP, collagen-1 (COL-1), RUNX2*, lipoprotein lipase (*LPL*), and peroxisome proliferator-activated receptor-γ *(PPAR-γ*), were evaluated using qRT-PCR, as previously described (Ding et al., 2013). PCR primer sequences are shown in **Table 1**.

**Table 1:** Primers used for RT-qPCR.

Gene	Forward primer sequence (5′–3′)	Reverse primer sequence (5′–3′)
*ALP*	AGGCTTCTTCTTGCTGGTGG	AGAGTGTCTTCCGAGGAGGTCA
*COL-1*	AGACGAAGACATCCCACCAATC	GATCACGTCATCGCACAACAC
*RUNX2*	CGGAATGCCTCTGCTGTTATG	AAGGTGAAACTCTTGCCTCGTC
*LPL*	CAGGATGTGGCCCGGTTTAT	CGGGGCTTCTGCATACTCAA
*PPAR-γ*	TCGAGGACACCGGAGAGG	CACGGAGCTGATCCCAAAGT

### Western blotting

Total protein was extracted from the samples using lysis buffer (Beyotime), and protein concentrations were measured using a bicinchoninic acid (BCA) protein assay kit (Tiangen, China) according to the manufacturer’s instructions. Subsequently, 20 μg of total protein from each sample was separated using 10% sodium dodecyl sulfate-polyacrylamide gel electrophoresis (SDS-PAGE) and transferred to a polyvinylidene fluoride (PVDF) membrane. After 2 h, the membranes were blocked using 5% nonfat milk for 1 h and incubated with primary antibodies against RUNX2 (Cell Signaling, #12556, rabbit), osteocalcin (Fonseca-Pereira et al., 2014) (Santa Cruz Biotechnology, sc-390877, mouse), and actin (Cwbio, CW0096A, mouse) overnight at 4°C. The following day, the membranes were washed with Tris-buffered saline tween (TBST) and incubated with appropriate secondary antibodies for 1 h at room temperature. Finally, the blots were visualized using a Western-light chemiluminescent detection system (Tanon-Bio, Shanghai, China).

### Characterization of the Lyso-PEG emulsion

Lyso-PEG emulsion was prepared according to a previously described method (Li et al., 2018; Yang et al., 2022). The Lyso-PEG emulsion (100 μl) was diluted to 5 ml in ultrapure water, and a copper grid (supported carbon film) was then immersed in the diluted sample and removed after 15 min. The liquid drop was then absorbed with filter paper, and the sample was further negatively stained with a 1% (w/v) aqueous solution of phosphotungstic acid (pH 7.0) for 1 min and then washed with ultrapure water for 45 s. The samples were then dried in air at room temperature and analyzed using transmission electron microscopy (TEM; HT-7700; Tokyo, Japan). The size distribution of the Lyso-PEG colloids in the emulsion was determined using a Zetasizer Nano S (ZEN 3600, Malvern, England) at predetermined time points (days 0, 4, 8, 12, and 16).

Fourth-generation SHED was cultured at an initial seeding density of 5000 cells cm^-2^ on Lyso-PEG-coated round coverslips. After 24 h, the samples were washed with PBS and fixed with 4% paraformaldehyde for 20 min at room temperature. Cell proliferation was analyzed using the CCK-8 assay after culturing the different groups for 1–7 d.

### Lyso-PEG as a matrix for tooth slabs remineralization

The tooth slabs of deciduous teeth from healthy individuals and patients with CCD (prepared as previously described, cleaned ultrasonically) were separately immersed in the Lyso-PEG emulsion, followed by the application of a small cotton ball dipped in solution for 10 min. After treatment, the samples were dried in air, and then, samples with a Lyso-PEG nanofilm coating were immersed in artificial saliva (10 ml per sample; containing 1.5 mM CaCl_2_, 0.9 mM K_2_HPO_4_, 1.5 ppm F^−^, 130 mM KCl, and 20 mM HEPES buffer at pH 7.00 ± 0.03) in a 37°C thermotank and collected after 48 h. The samples were then washed with ultrapure water and dried under vacuum. Finally, the dentin structure and morphology were analyzed using SEM and EDX, as described above.

### Statistical analysis

All data are expressed as mean ± standard deviation (SD) of at least three independent experiments. Statistical significance between two groups was calculated using the Student’s *t*-test (two-tailed). GraphPad Prism 8.0 was used to perform statistical analysis and prepare graphs. Statistical significance was set at p < 0.05 (*p < 0.05; **p < 0.01; and ***p < 0.001).

## Results

### Typical clinical and radiological findings in patients with CCD with novel missense mutation

**Figure 1:**
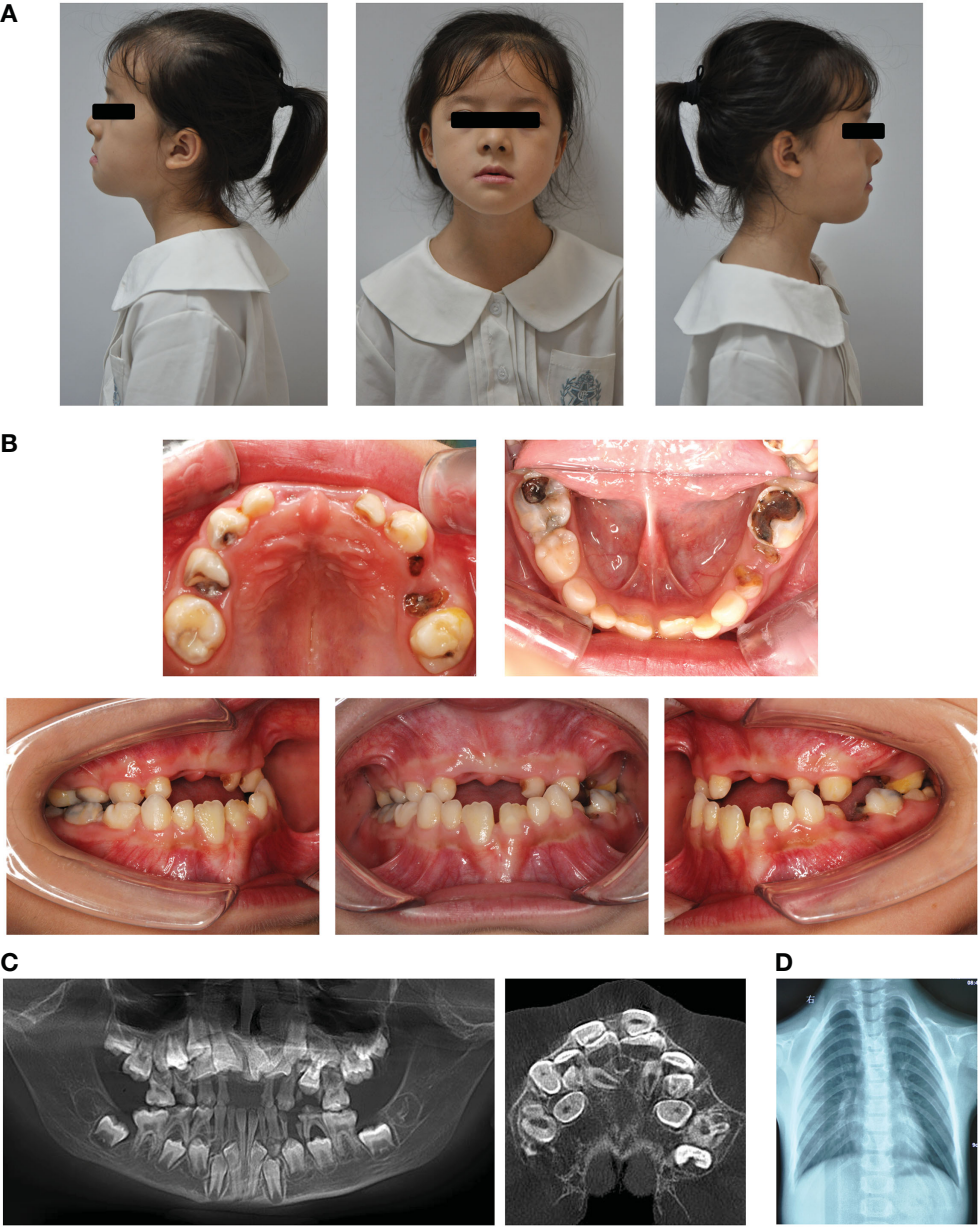
Typical clinical and radiological findings in the proband with CCD. **(A)** Front and profile photographs of the individual showing a concave face, hypoplasia of the maxilla, and a depressed nasal bridge. **(B)** Intraoral images showing enamel and dentin hypoplasia. **(C)** Panoramic view showing embedded permanent, supernumerary, and retention of deciduous teeth. **(D)** Chest radiograph showing hypoplasia of the left clavicle.

**Figure 2:**
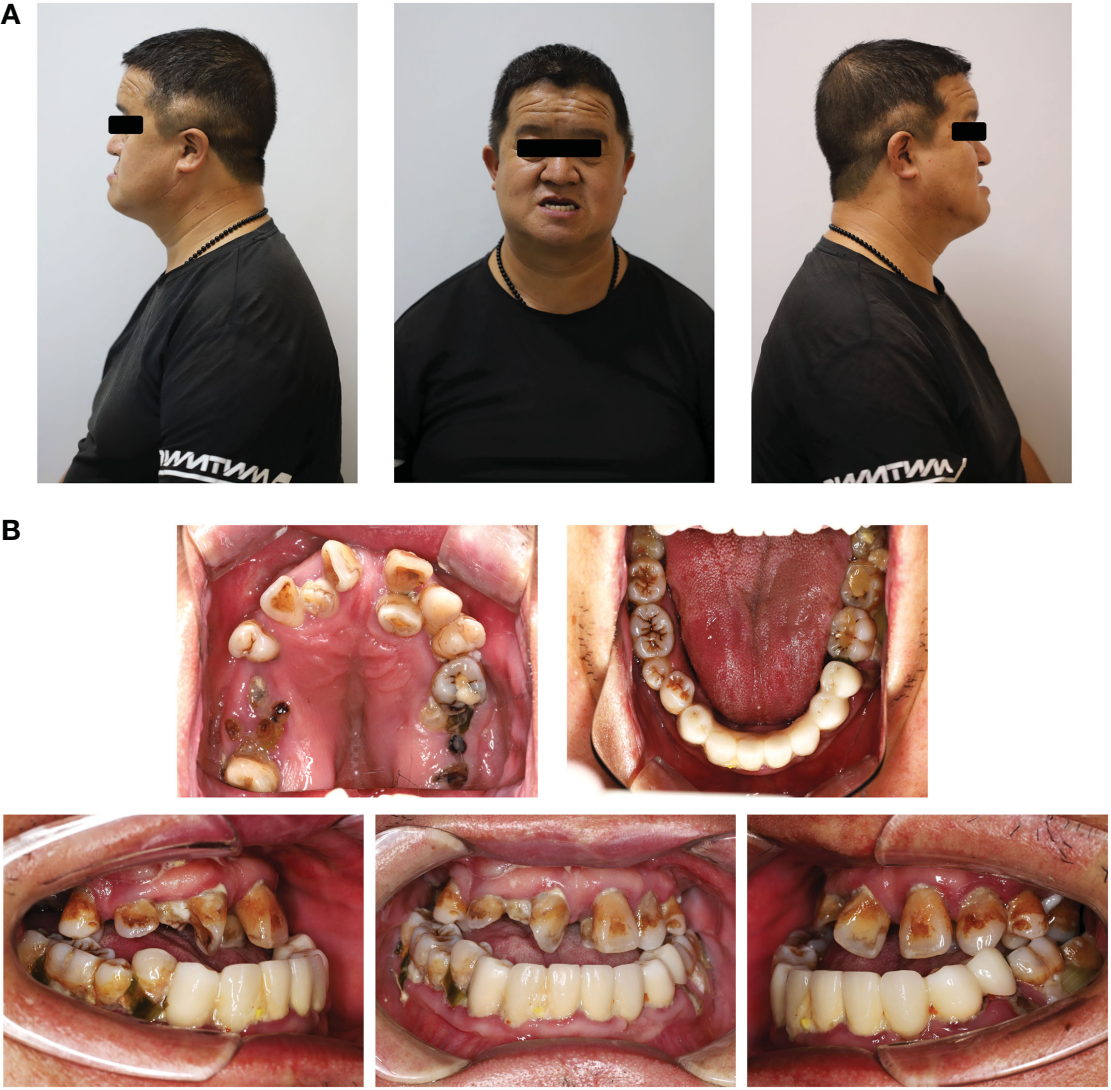
Typical clinical and radiological findings in the father of the proband. **(A)** Front and profile photograph of the individual showing a concave face, hypoplasia of the maxilla, and a depressed nasal bridge. **(B)** Intraoral image showing enamel and dentin hypoplasia and the lower anterior teeth missing.

**Figure 3:**
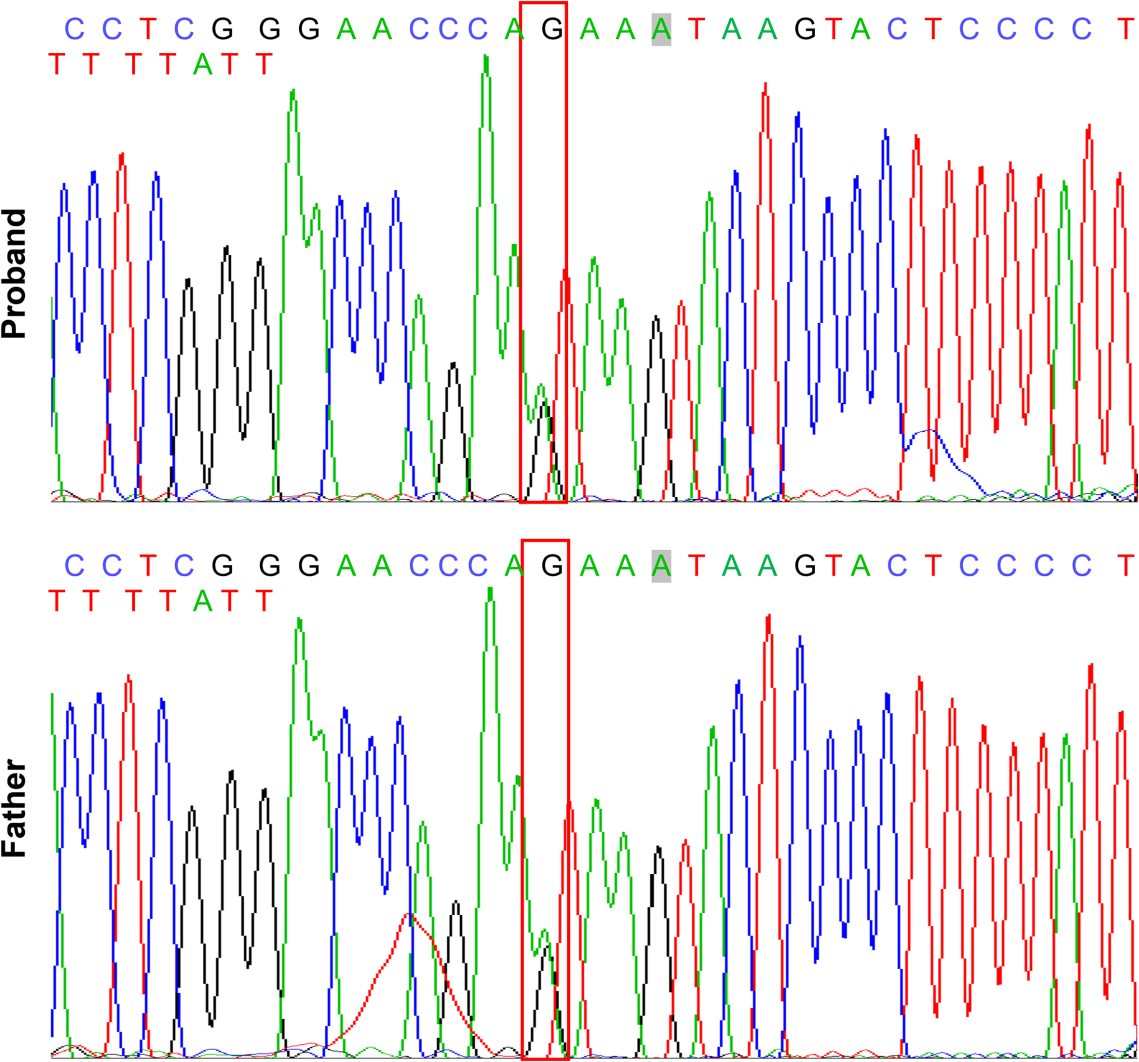
Mutation analysis of *RUNX2* gene in the patients with CCD. Sequencing results showing the heterozygous mutant of *RUNX2* (c. 685 + 1G>A, p. Arg228Asp). The box indicates the mutation site.

Clinical examination showed that the 8-year-old proband had narrow shoulders, a concave face, and a depressed nasal bridge (**Figure 1A**). Intraoral examination revealed extensive caries in 54, 53, 75, and 85 teeth. In addition, 64 and 74 teeth showed residual roots, respectively (**Figure 1B**). Panoramic radiography revealed delayed eruption of the permanent dentition and supernumerary teeth (**Figure 1C**). Chest radiography showed hypoplasia of the left clavicle (**Figure 1D**). Typical clinical and radiological findings indicated a clinical diagnosis of CCD. Additionally, the proband’s father had narrow shoulders, a concave face, and a depressed nasal bridge (**Figure 2A**). Intraoral examination revealed residual roots in 15, 16, 26, and 27 teeth (**Figure 2B**). Similar severe multiple teeth decay occurred in both father and daughter, indicating hypoplasia of the tooth. Genomic DNA was extracted from 5 ml of peripheral blood leukocytes from the proband and her father. Human whole genome sequencing (WGS) revealed that the proband and her father were heterozygous for a novel missense mutation c.685+1G>A (p. Arg228Asp), in *RUNX2* (NM_001024630.4), which may be responsible for CDD (**Figure 3**). These findings provide further information about the clinical features and gene mutations of CCD.

### Dentinogenesis imperfecta of CCD

**Figure 4:**
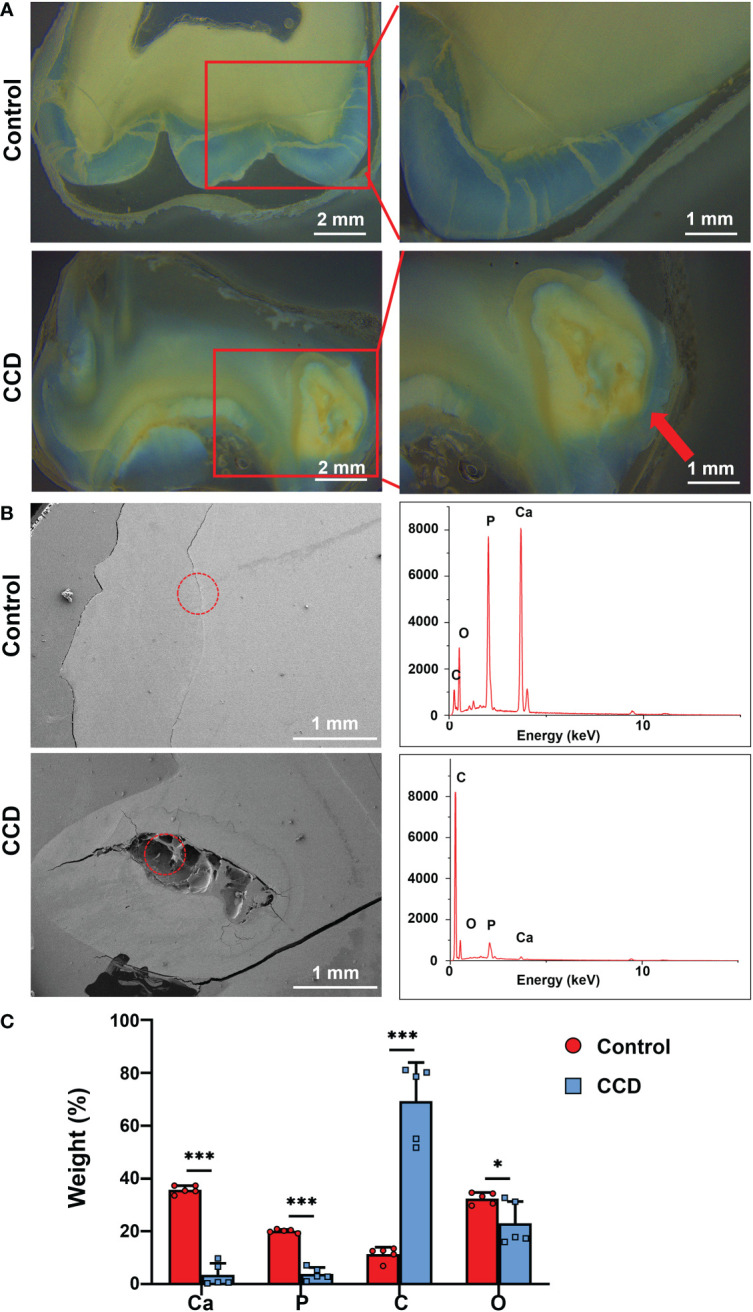
Dentinogenesis imperfecta of patients with CCD. **(A)** Stereo microscopy showing the morphology difference between the two groups. Red arrow indicates the disorganized structure of dentin. Scale bar: 2 mm, 1 mm. **(B)** SEM images and EDX spectra of the two groups. The red circle refers to the analyzed area of the EDX spectra. Scale bar, 1 mm. **(C)** Weight percentage of the elements of tooth slabs of the two groups (n = 5, *p < 0.05, ***p < 0.001).

The retained deciduous teeth of the proband were extracted and collected for morphological and structural analyses. Due to enamel exfoliation and decay, this study only evaluated the dentin structure. Stereo microscopy showed the disorganized morphology of dentin in the CCD tooth slabs (**Figure 4A**). In addition, the SEM images showed noteworthy defects near the enamel-dentinal junction in the CCD group. EDX analysis showed a remarkable reduction in calcium and phosphorus in the defect area in the CCD group compared with the control group in the enamel-dentinal junction area (**Figure 4B**). Finally, the dentin areas of both the control and CCD groups were selected for the EDX analysis. Compared to the control group, the EDX spectra exhibited a remarkable reduction in the weight percent of calcium and phosphorus, and an obvious increase in carbon in the CCD group (*p < 0.05 and ***p < 0.001) (**Figure 4C**). These findings indicate that the patients with CCD suffered enamel exfoliation and dentinogenesis imperfecta, which may be responsible for their early extensive caries and tooth loss.

### The SHED from CCD exhibited impaired proliferative property

**Figure 5:**
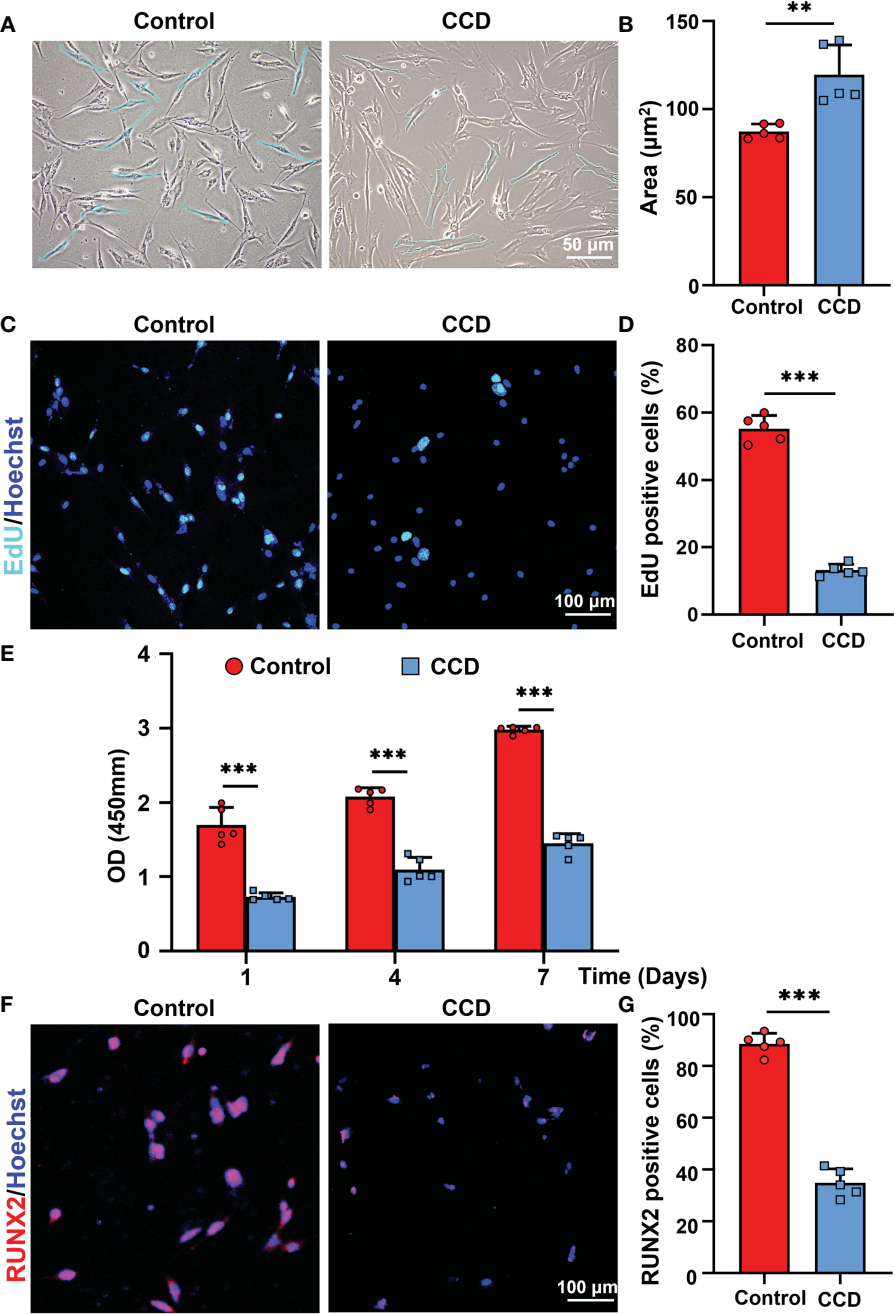
The SHED from CCD exhibits impaired proliferative property. **(A)** Optical microscopy showing the morphology and size of the fourth-generation cells. Scale bar: 50 μm. **(B)** The bar graph illustrates the areas of cells (n = 5, **p < 0.01). **(C)** CLSM showing the results of cell proliferation of SHED. Scale bar: 100 μm (n = 5, ***P < 0.001). **(D)** The bar graph illustrates the proportion of EdU positive cells (n = 5, ***p < 0.001). **(E)** Cell viability of SHED cultured after 1, 4 and 7 d (n = 5, ***p < 0.001). **(F)** RUNX2 expression on the SHED from the two groups. Scale bar: 100 μm. **(G)** The bar graph illustrates the percentage of RUNX2 positive cells (n = 5, ***p < 0.001).

Because of the dentinogenesis imperfecta and *RUNX2* mutation in the CCD group, the SHED from the control and CCD groups were collected to analyze the cell biological properties. Optical microscopy showed that the morphology of SHED in the CCD group appeared larger and fatter than that in the control group, lacking the characterized spindle cell morphology of MSCs (**Figure 5A**). The area of SHED in the CCD group was larger than that in control group (**p < 0.01) (**Figure 5B**). The EdU assay demonstrated a distinct reduction in the number of proliferative cells in the CCD group (***p < 0.001) (**Figures 5C, D**). Furthermore, the CCK-8 assay showed that both groups possessed proliferative potential, but CCD revealed lower cell proliferation after 1, 4, and 7 d compared to the control group (***p < 0.001) (**Figure 5E**). These findings demonstrated that SHED from the CCD group possessed impaired proliferative properties. To further examine the protein expression of RUNX2 in cells from the two groups, immunofluorescence was performed. In the control group, RUNX2 was found to be expressed in the nucleus. In the CCD group, which had a heterozygous mutation of *RUNX2*, the percentage of RUNX2-positive cells decreased distinctly compared to that in the control group (***p < 0.001) (**Figures 5F, G**). These findings indicate that mutation of *RUNX2* may lead to impaired proliferative properties of SHED derived from CCD.

### The impaired osteogenic potential of SHED from CCD

**Figure 6:**
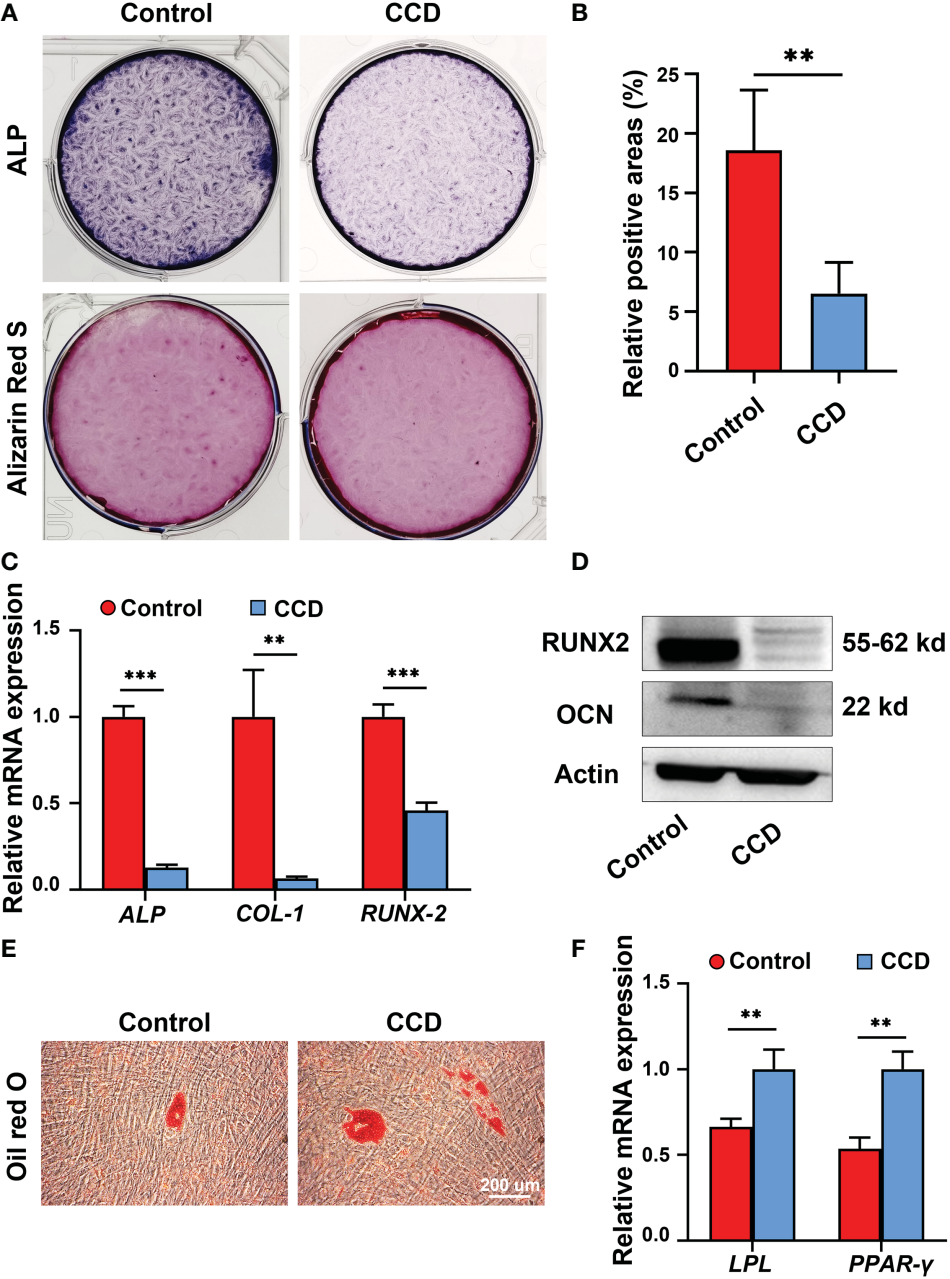
The impaired osteogenic potential of SHED from patients with CCD. **(A)** ALP and Alizarin Red S staining of SHED after osteogenic induction. **(B)** Alizarin Red S staining showing mineralized nodules obviously decreased in CCD group compared with the control group (n = 5, **p < 0.01). **(C)** qRT-PCR analysis of the expression of *ALP, COL-1*, and *RUNX2* after 14 d of osteogenic induction (n = 5, **p < 0.01, ***p < 0.001). **(D)** Western blot analysis showing OCN and RUNX2 protein expression levels in the two groups. Scale bar: 200 μm. **(E)** Oil red O staining of SHED after 7 d of adipogeneic induction. **(F)** qRT-PCR analysis of the expression of *LPL* and *PPAR-γ* of SHED (n = 5, **p < 0.01).

To further analyze the influence of heterozygous mutation of *RUNX2* on SHED from CCD, an osteogenic potential assay alkaline phosphatase (ALP) staining and Alizarin Red S staining were performed. The results showed that both groups possessed osteogenic potential, whereas the ALP expression level and mineralized nodules obviously decreased in the CCD group compared to the control group (**p < 0.01) (**Figures 6A, B**). Additionally, the mRNA expression of the osteogenic genes *ALP, COL-1* and *RUNX2* was determined using qRT-PCR. The findings exhibited lower osteogenic genes expression levels in the CCD group than in the control group after 14 d of osteogenic induction (**p < 0.01, and ***p < 0.001) (**Figure 6C**). The protein expression levels of the osteogenic proteins RUNX2 and OCN were assessed using Western blotting. Compared to the control group, RUNX2 and OCN of the CCD group exhibited lower protein expression levels after 14 d of osteogenic induction (**Figure 6D**). Finally, Oil Red O Staining was performed to detect the adipogenic differentiation capacity of the two groups. The findings showed that the CCD group possessed higher adipogenesis potential than the control group, with more lipid droplet formation and higher expression levels of the adipogenic genes *LPL* and *PPAR-γ* (**p < 0.01) (**Figures 6E, F**). These findings reveal that SHED from the CCD group exhibited impaired osteogenic potential but enhanced adipogenic potential, implying that the mutant of *RUNX2* may affect the osteogenic potential of SHED.

### Characteristics of Lyso-PEG emulsion

**Figure 7:**
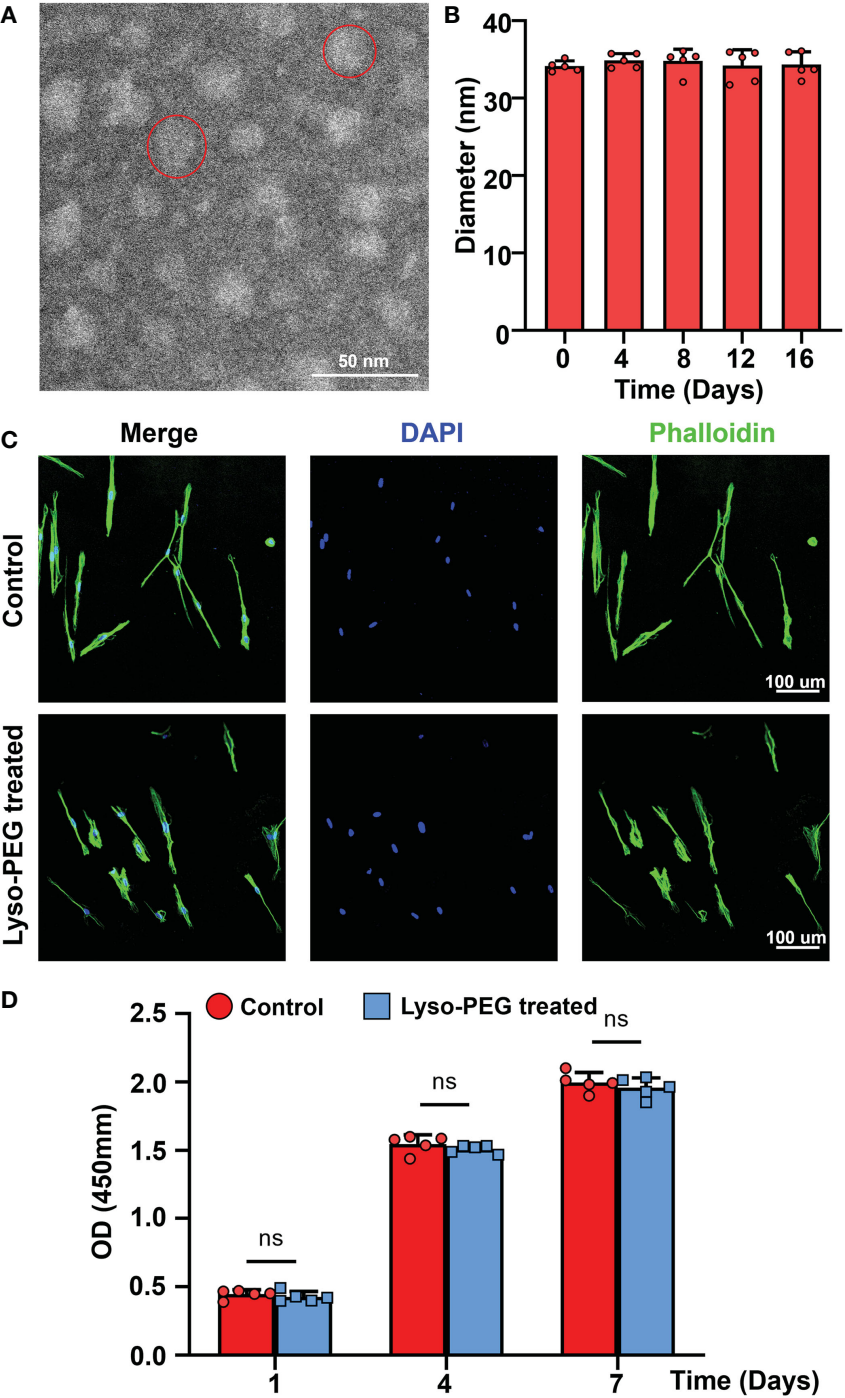
Characteristics of Lyso-PEG emulsion. **(A)** TEM image of the Lyso-PEG oligomeric nanoparticles; the red circle indicates an oligomeric nanoparticle. Scale bar: 50 nm. **(B)** The diameter of the Lyso-PEG emulsion for 20 d at room temperature (n = 5). **(C)** CLSM image of SHED on the round coverslip. Scale bar: 100 μm. **(D)** Cell viability of SHED after 1, 4, and 7 d (n = 5, ^ns^p > 0.05).

To further investigate the biological safety of the Lyso-PEG emulsion on SHED, Lyso-PEG emulsion and SHED from healthy individuals were co-cultured for 24 h. SHED cultured without the Lyso-PEG emulsion was used as a control. First, TEM imaging showed that the Lyso-PEG oligomer nanoparticle were round and approximately 30 nm in size in the solution (**Figure 7A**). Diameter analysis showed that the particle size remained stable without obvious aggregation for at least 20 d (**Figure 7B**). With regard to the biosafety of the Lyso-PEG emulsion, the CLSM results showed that after co-culture with Lyso-PEG, the cell morphology and structure showed no clear difference from those of the control (**Figure 7C**). The CCK-8 assay was used to evaluate the cytotoxicity of the Lyso-PEG emulsion. The results showed that after co-culturing with Lyso-PEG, there was a similar proliferative property between the two groups at different time points (**Figure 7D**). These findings showed the ideal biocompatibility of Lyso-PEG.

### Lyso-PEG as a matrix for dentin remineralization of CCD

**Figure 8:**
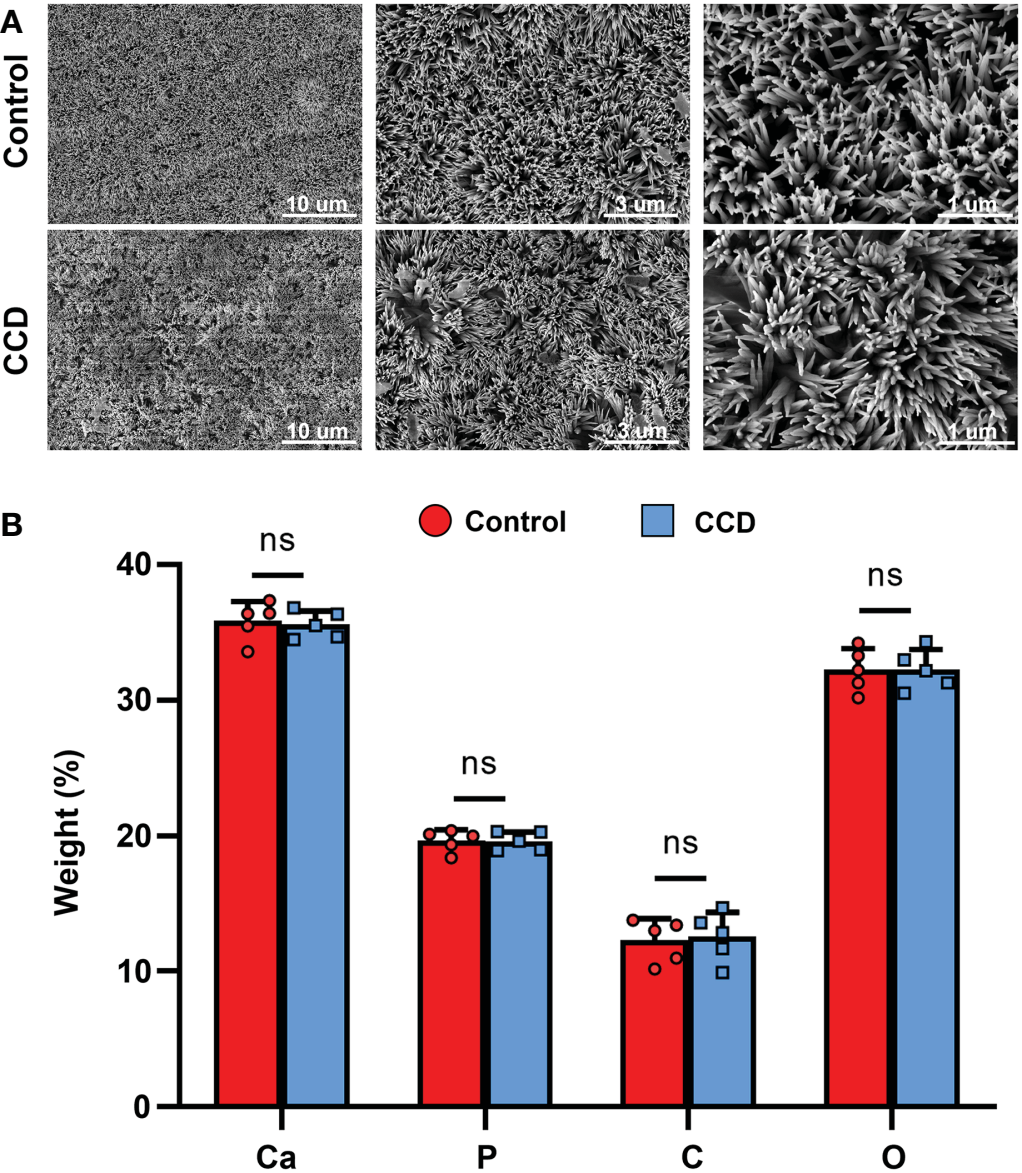
Lyso-PEG as a matrix for dentin remineralization of patients with CCD. **(A)** SEM images of Lyso-PEG-induced remineralization structures on dentin of the two groups. Scale bars: 10, 5, and 1 μm. **(B)** Weight percentage of the elements of tooth slabs in two groups (n = 5, ^ns^p > 0.05).

The morphology of the tooth slabs treated with the Lyso-PEG emulsion was first examined using SEM, with healthy tooth slabs as the control group. After treatment with Lyso-PEG under the same conditions, a dense, well-organized, and newly formed layer was observed in both groups (**Figure 8A**). When the dentin surface was analyzed using EDX, the results showed that the main elements on the surface of the two groups were similar, and the Ca/P ratio of the two groups was similar, which was close to that of the natural tooth slab (**Figure 8B**). These findings indicate that Lyso-PEG possess favorable remineralization capacity in both healthy and dysplastic dentin, providing a promising strategy for dental caries prevention for patients with CCD or dentinogenic imperfecta.

## Discussion

This study identified a family with CCD with a novel missense mutation within *RUNX2* (c. 685 + 1G>A, p. Arg228Asp). The dentin structure and SHED from CCD and healthy individuals were evaluated and compared, and it was found that CCD in this study displayed hypoplasia of dentin, as well as impaired proliferative and osteogenic potential of SHED. To enhance the caries-preventive capacity of CCD, Lyso-PEG emulsion was applied to the tooth slab from healthy and CCD individuals, obtaining a similar mineralized layer on the dentin surface. This study provides more information about the dentin and SHED of CCD, providing a novel material, Lyso-PEG emulsion, for caries prevention in patients with CCD.

CCD is an autosomal-dominant heritable skeletal disease that affects cranial sutures, teeth, and clavicles. Typical clinical features include absent or hypoplastic clavicles, defective ossification of the anterior fontanelle, and supernumerary teeth (Sun et al., 2016; Zhang et al., 2022). In addition to clear systemic symptoms, dental anomalies such as skeletal class III tendency, high narrow arched palate, tooth hypoplasia, delayed eruption of permanent dentition and multiple impacted supernumerary teeth are characteristic abnormal oral manifestations, that severely affect the oral health of patients (Shih-Wei Cheng et al., 2022; Yamashiro et al., 2022). Since the delayed eruption of permanent dentition and arrest of multiple impacted supernumerary teeth in the replacement of primary and permanent teeth, studies on CCD have mainly focused on the mechanism of dental replacement (Qin et al., 2019; Xin et al., 2022; Zeng et al., 2022). However, the clinical features of severe caries and early tooth loss, which also influence the oral and mental health of individuals with CCD, have been received little attention. It has been reported that individuals with CCD exhibit enamel exfoliation and dentin dysplasia (Chang et al., 2017). In this study, the proband and her father both had caries in multiple teeth, and some caries even resulted in tooth loss (**Figures 1B**, **2B**). To further examine the tooth structure of the CCD, primary teeth from the proband were collected and sectioned into tooth slabs. Because of enamel exfoliation and decay, this study failed to obtain sufficient enamel samples for examination. SEM and EDX analyses revealed the disorganized structure and hypocalcification of dentin in the CCD group (**Figure 4**), indicating that dentinogenesis imperfecta of CCD led to early extensive dental caries and tooth loss.

*RUNX2* is a transcription factor that is crucial for bone and tooth formation, as well as for osteoblast and dental stem cells differentiation (Suo et al., 2020; Kim et al., 2020a). Heterozygous mutations in *RUNX2* may be the major cause of CCD (Sun et al., 2016). Studies in mice have shown that Runx2 exhibits a spatiotemporal expression pattern during early tooth development. Before the early bell stage, Runx2 is highly expressed in mesenchymal cells, which are a source of odontoblasts. In addition, *Runx2* homozygous null mice displayed arrested osteoblast differentiation and tooth development at the late bud stage (James et al., 2006; Kim et al., 2020b). However, there is still a lack of functional evidence regarding *RUNX2* mutations in patients with CCD. In this study, the proband and her father displayed the typical clinical features of CCD (**Figures 1**, **2**), with the same novel heterozygous missense mutation in *RUNX2* (c.685+1G>A, p. Arg228Asp) (**Figure 3**). These findings indicate that the *RUNX2* mutation may be responsible for CCD in this case. However, further efforts are required toward understanding the phenotype-genotype correlation.

The pulp-dentin complex plays a crucial role in maintaining tooth homeostasis. Dental pulp stem cells can differentiate into odontoblast-like cells, forming secondary or reparative dentin. (Chen et al., 2021; Rahman et al., 2022). Exploring the function of the pulp-dentin complex facilitates comprehensive identification of the underlying mechanism of dental abnormalities in CCD. SHED is a type of dental pulp stem cell obtained from deciduous teeth and possesses cell viability and regenerative properties (Xuan et al., 2018). Studies have reported that CCD-associated *RUNX2* mutations may reduce the proliferation and mineralization capacity of the SHED of the patient, along with their ability to induce odontoblast differentiation, which may be a cause of dental abnormalities in these patients (Xuan et al., 2010; Yan et al., 2015; Greene et al., 2018). Delayed eruption of the permanent dentition and supernumerary teeth are the most common oral manifestations of CCD (Komori, 2020). As *RUNX2* is the causative gene for CCD and is important in tooth formation, different types of cells with heterozygous mutations of *RUNX2* show diverse dysfunction, leading to various oral manifestations when mutated. For instance, dental follicle cells with *RUNX2* mutations show weaker osteogenesis and osteoclastic ability, resulting in tooth eruption and alveolar bone remodeling difficulties, and periodontal ligament cells with *RUNX2* mutations demonstrate an affected balance between osteoblast and osteoclast activity, leading to tooth eruption difficulties and deciduous tooth retention (Komori et al., 1997; Sun et al., 2016; Zeng et al., 2022). In the present study, SHED obtained from the proband showed a weaker capacity for proliferation and osteogenesis than SHED obtained from healthy individuals (**Figures 5**, **6**), consistent with previous reports. Notably, the percentage of RUNX2 expression decreased in SHED from patients with CCD, which was consistent with the results of the missense mutation in *RUNX2* (**Figures 5F, G**). These findings extend our understanding of CCD and further uncovered the influence of *RUNX2* mutation on SHED.

Previously, our research team identified a new strategy for enamel remineralization through amyloid-mediated biomimetic remineralization of Lyso-PEG, achieving an ideal enamel-like crystalline hydroxyapatite layer on healthy human tooth pits and fissures (Yang et al., 2022). The disulfide bonds of lysozyme can be reduced by tris (2-carboxyethyl) phosphine (TCEP), forming nanoscale phase-transited lysozyme (PTL) oligomeric aggregates, which is similar to amyloid-like aggregation. The PTL oligomeric aggregates conjugated with PEG, referred to as Lyso-PEG, can form a stable emulsion without aggregation for at least 20 d at room temperature (Wang et al., 2016; Li et al., 2018). However, whether Lyso-PEG is safe for SHED remains elusive. In this study, the findings of the CCK-8 test and immunofluorescence staining of the morphology and structure of cells demonstrated that Lyso-PEG showed ideal biological compatibility with SHED and can be used clinically (**Figure 7**). Although SHED in this experiment was applied to evaluate the biological safety of Lyso-PEG, whether SHED from patients with CCD show similar biological compatibility needs further investigation.

It has been reported that a Lyso-PEG emulsion can form a stable nanofilm on the tooth enamel surface (Liu et al., 2021). The amyloid-like aggregation of Lyso-PEG has been shown to form a biomimetic mineralization matrix (Zhang et al., 2020), which is full of carboxyl, amine, and hydroxyl groups, thus inducing the formation of a mineralized structure by absorbing ions from saliva and mediating *in situ* nucleation and growth of crystalline hydroxyapatite (Yang et al., 2022). However, whether Lyso-PEG emulsion induces dentin remineralization remains unclear. In the present study, after Lyso-PEG was applied to the dentin surface of the two groups, the CCD and control groups both formed a similar nanofilm coated structure, indicating that the Lyso-PEG emulsion possessed ideal remineralization properties to prevent tooth hypoplasia (**Figure 8**). However, further investigation is required to elucidate its clinical efficacy and underlying mechanisms.

In conclusion, the present study presented a family with CCD with a novel missense mutation in RUNX2. The proband exhibited hypoplasia of dentin as well as impaired proliferative and osteogenic potential of SHED. The study first found that the Lyso-PEG emulsion directly formed an ideal nanofilm on dentin, providing a promising insight into caries prevention in patients with CCD or dentin dysplasia.

## Data availability statement

The original contributions presented in the study are included in the article/Supplementary Materials. Further inquiries can be directed to the corresponding authors.

## Ethics statement

The studies involving human participants were reviewed and approved by The Institutional Review Board and the Ethics Committee of the Fourth Military Medical University (FMMU). Written informed consent to participate in this study was provided by the participants’ legal guardian/next of kin. Written informed consent was obtained from the individual(s), and minor(s)’ legal guardian/next of kin, for the publication of any potentially identifiable images or data included in this article.

## Author contributions

XG and XY wrote the manuscript with comments from all authors. XY, HG, and PL performed the biological experiments and evaluated material efficacy *in vitro*. XH and YG analyzed data. AL and KX supervised the experiments, interpreted results, and provided critical revisions of the manuscript. All authors read and approved the final manuscript. All authors contributed to the article and approved the submitted version.
